# GSG2 promotes thyroid cancer via stabilizing AURKB and activating AKT pathway

**DOI:** 10.18632/aging.205605

**Published:** 2024-03-04

**Authors:** Fenghua Zhang, Chiming Huang

**Affiliations:** 1 Department of Thyroid and Breast Surgery, Hebei General Hospital, Shijiazhuang 050051, Hebei Province, China; 2 Thyroid Hernia Surgery, Guangdong Provincial People’s Hospital, Guangdong Academy of Medical Sciences, Yuexiu, Guangzhou 510182, Guangdong Province, China

**Keywords:** thyroid cancer, GSG2, AURKB, AKT pathway, cell phenotypes

## Abstract

Thyroid cancer stands out as the most prevalent endocrine cancer, with its incidence on a global rise. While numerous studies have delved into the roles of GSG2 in the progression of various malignancies, its involvement in thyroid cancer remains relatively unexplored. Therefore, this study was initiated to assess the functional importance of GSG2 in human thyroid cancer development. Our findings revealed a notable upregulation of GSG2 in both thyroid cancer tissues and cell lines, demonstrating a significant correlation with the pathological stage and patients’ prognosis. Depletion of GSG2 in thyroid cancer cells resulted in suppressed malignant cell development and inhibited tumor outgrowth. Crucially, our investigation identified AURKB as a downstream gene of GSG2. GSG2 exhibited its regulatory role by stabilizing AURKB, countering SMURF1-mediated ubiquitination of AURKB. Furthermore, overexpressing AURKB restored the functional consequences of GSG2 depletion in thyroid cancer cells. Additionally, we proposed the involvement of the AKT pathway in GSG2-mediated regulation of thyroid cancer. Intriguingly, the reversal of cell phenotype alterations in GSG2-depleted cells following an AKT activator underscored the potential link between GSG2 and the AKT pathway. At the molecular level, GSG2 knockdown downregulated p-AKT, an effect partially restored after AKT activator treatment. In summary, our study concluded that GSG2 played a pivotal role in thyroid carcinogenesis, underscoring its potential as a therapeutic target for thyroid cancer.

## INTRODUCTION

Thyroid cancer, though rare, accounts for less than 1% of human neoplasms; however, it frequently manifests in the endocrine system and is responsible for the majority of endocrine cancer-related deaths [1]. The global incidence of thyroid cancer continues to rise [2]. Originating from follicular epithelial cells or parafollicular C cells, thyroid cancer is histologically categorized into well-differentiated thyroid cancers (WDTCs), poorly differentiated thyroid cancer (PDTC), and anaplastic thyroid cancer (ATC) [3–5]. Notably, WDTCs represent the predominant contributor to thyroid cancer-related mortality [6]. For individuals with low-risk WDTC, surgery stands as the primary therapeutic approach [7]. However, for patients grappling with advanced and metastatic thyroid cancer, although targeted therapies have shown promise in extending overall survival, the prognosis remains poor [8–11]. Hence, there is an imperative need to explore additional therapeutic targets to enhance our understanding and management of thyroid cancer.

GSG2, also known as Histone H3 associated protein kinase (Haspin), was initially discovered in the mouse testis gene [12]. As a serine/threonine kinase, GSG2 plays a pivotal role in the cell cycle of eukaryotic cells by specifically phosphorylating H3T3 on nucleosomes [13]. During mitosis, the phosphorylation of H3T3 by GSG2 enables the localization of Aurora B at the centromere. Subsequently, the activation of Aurora B by recognizing H3T3ph facilitates the proper arrangement of chromosomes, thereby promoting the cell cycle [14]. A growing body of evidence has highlighted the association between GSG2 and the pathogenesis of various cancers. Notably, the development of CHR-6494, a small molecule inhibitor targeting GSG2, has shown promising outcomes in combatting breast, colon, and cervical cancer [15, 16]. Building upon this knowledge, we postulated that GSG2 may also play a role in the development of thyroid cancer.

Hence, the primary objective of the present study is to explore the expression levels, functional significance, and molecular mechanisms of GSG2 in thyroid cancer development. Through this investigation, we aim to assess the potential of GSG2 as a therapeutic target for thyroid cancer.

## RESULTS

### Expression of GSG2 in thyroid cancer tissues and cells

**Figure 1:**
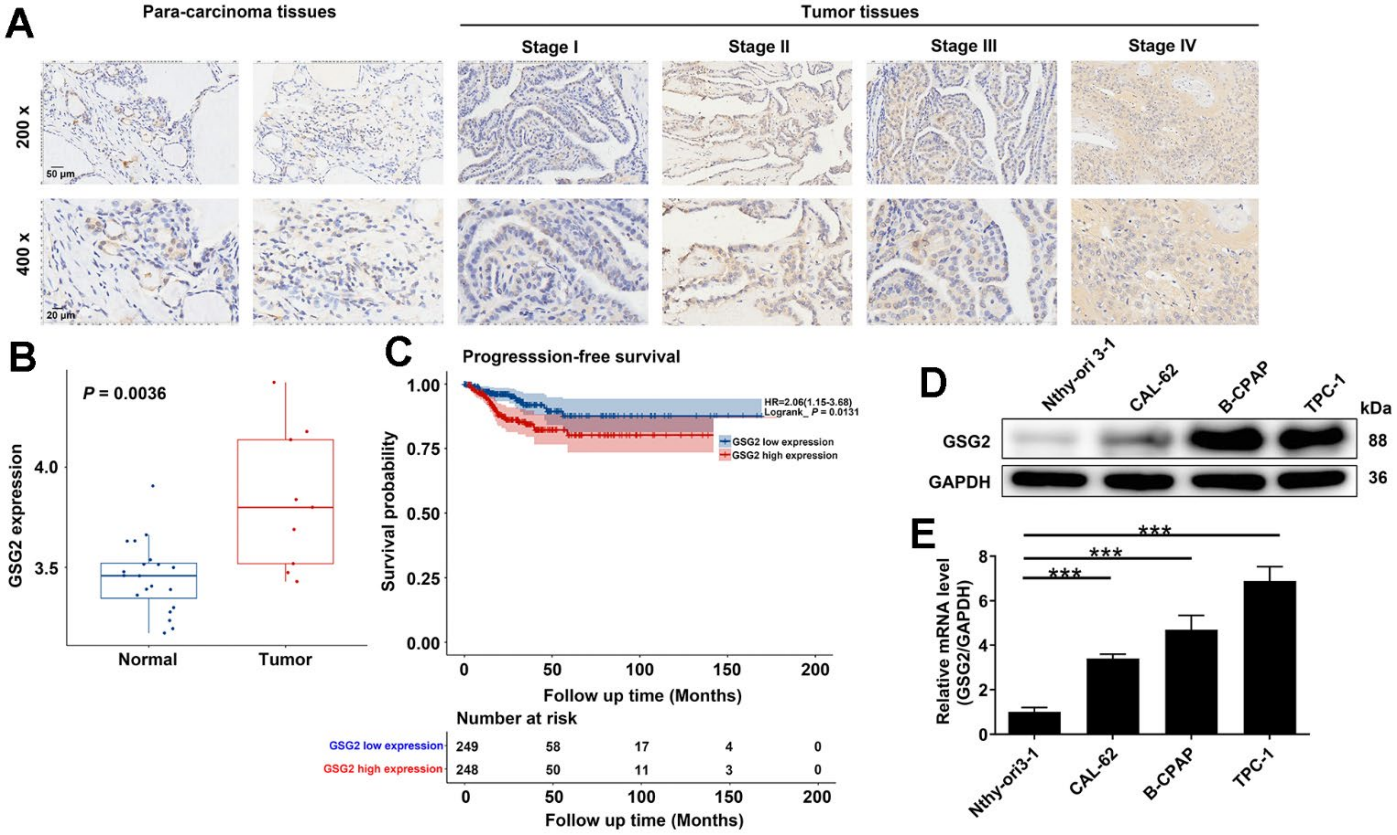
**Expression of GSG2 in thyroid cancer tissues and cells.** (**A**) Representative images of IHC analysis on GSG2 protein expression in thyroid cancer tissues compared to para-carcinoma tissues. (**B**) Differential expression of GSG2 in thyroid cancer compared to normal tissues based on analysis of the GSE29265 database. (**C**) Prognostic analysis of GSG2 expression in thyroid cancer samples from the TCGA database. (**D**, **E**) GSG2 mRNA and protein levels in Nthy-ori 3-1 and thyroid cancer cell lines (CAL-62, B-CPAP, TPC-1) were detected through western blot (**D**) and qRT-PCR analysis (**E**). Results were presented as mean ± SD. *** *P* < 0.001.

Firstly, our investigation delved into the expression levels of GSG2 in thyroid cancer. IHC analysis conducted on a tissue microarray from 40 thyroid cancer patients revealed a significant elevation of GSG2 protein in thyroid cancer tissues compared to adjacent normal tissues (*P* < 0.001, Figure 1A and Table 1). Furthermore, Tables 2, 3 outlined the correlation between GSG2 levels and clinicopathological characteristics of the patients. Notably, high-stage thyroid cancer exhibited higher GSG2 expression than low-stage thyroid cancer. However, no significant associations were observed between GSG2 expression and other indicators, such as age, gender, and tumor infiltrate. We also explored the differential expression of GSG2 in thyroid cancer using the GSE29265 database. The analysis revealed a significant upregulation of GSG2 expression in thyroid cancer compared to normal tissues (*P* < 0.01, Figure 1B). To assess the prognostic significance of GSG2, an analysis was conducted on thyroid cancer samples from the TCGA database. The results indicated a significant association between elevated GSG2 expression and notably shorter progression-free survival (PFS) compared to the low expression group (*P* < 0.05, Figure 1C). Furthermore, we compared the levels of GSG2 in Nthy-ori 3-1 and thyroid cancer cell lines (CAL-62, B-CPAP, and TPC-1 cells) using qRT-PCR and western blot experiments. The results highlighted a significant increase in both mRNA and protein levels of GSG2 in thyroid cancer cell lines relative to Nthy-ori 3-1 cells. This upregulation was particularly pronounced in B-CPAP and TPC-1 cells (Figure 1D, 1E). This comprehensive analysis provided a detailed understanding of the expression patterns and prognostic implications of GSG2 in thyroid cancer, shedding light on its potential role in the development of the disease.

**Table 1:** Expression patterns of GSG2 in thyroid cancer tissues and para-carcinoma tissues revealed in immunohistochemistry analysis.

**GSG2 expression**	**Tumor tissue**		**Para-carcinoma tissue**		***P*-value**
	**Cases**	**Percentage**	**Cases**	**Percentage**	
Low	20	50.0%	40	100%	< 0.001
High	20	50.0%	0	-	

**Table 2:** Relationship between GSG2 expression and tumor characteristics in patients with thyroid cancer.

**Features**	**No. of patients**	**GSG2 expression**		***P-*value**
		**Low**	**High**	
All patients	40	20	20	
Age (years)				0.348
< 40	19	11	8	
≥ 40	21	9	12	
Gender				1.000
Male	8	4	4	
Female	32	16	16	
Tumor infiltrate				0.355
T1	2	2	0	
T2	24	11	13	
T3	10	6	4	
T4	3	0	3	
Stage				0.023
I	24	15	9	
II	9	3	6	
III	3	1	2	
IV	3	0	3	

**Table 3:** Relationship between GSG2 expression and pathological stage in patients with thyroid cancer.

		**GSG2**
Stage	Spearman correlation	0.370
	Signification (double-tailed)	0.020
	N	39

### The effects of depleting GSG2 on thyroid cancer *in vitro* and *in vivo*

To assess the impacts of GSG2 knockdown on thyroid cancer cell functions, we utilized two RNA interference plasmids, namely shGSG2-1 and shGSG2-2, both designed to target GSG2. The efficiency of knockdown was evaluated through qRT-PCR and western blot analyses, revealing a significant decrease in GSG2 mRNA and protein levels upon transfection of shGSG2-1 and shGSG2-2 in B-CPAP and TPC-1 cells. Notably, the knockdown efficiency was more pronounced in the shGSG2-1 group compared to the shGSG2-2 group (Supplementary Figure 1A, 1B). To further validate the specificity of GSG2 knockdown, we investigated the expression levels of GSG1 following the depletion of GSG2. qRT-PCR and western blot analyses in B-CPAP and TPC-1 cells transfected with shGSG2-1 and shGSG2-2 revealed no significant changes in GSG1 levels (Supplementary Figure 1A, 1B). This indicated that the knockdown of GSG2 did not affect the expression of GSG1, providing additional evidence supporting the specificity and target selectivity of shGSG2-1 and shGSG2-2.

**Figure 2:**
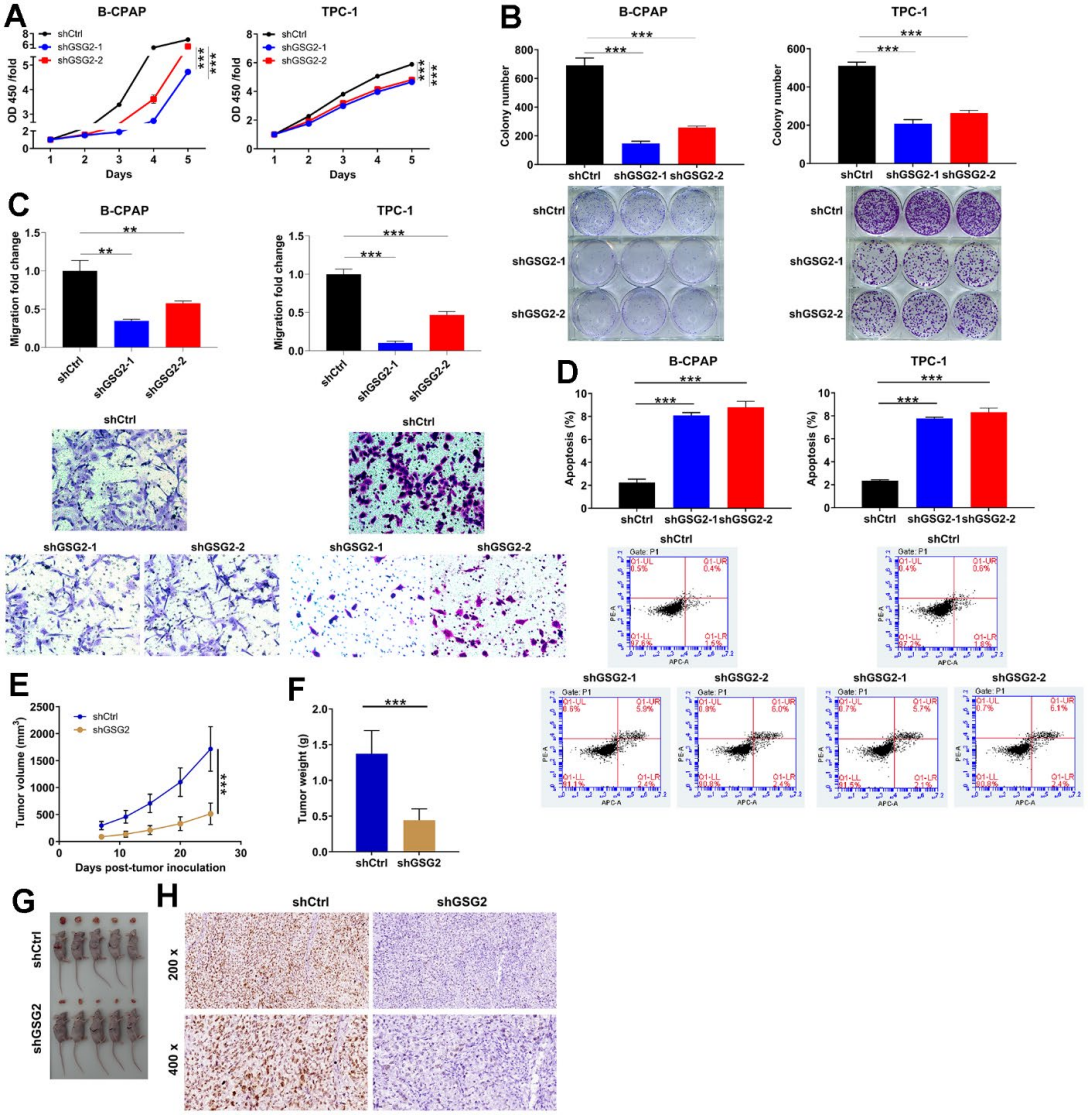
**The effects of depleting GSG2 on thyroid cancer *in vitro* and *in vivo*.** (**A**) CCK8 assay demonstrating a significant reduction in proliferation of B-CPAP and TPC-1 cells upon shGSG2-1 and shGSG2-2 transfection. (**B**) After shGSG2-1 and shGSG2-2 transfection, Colony formation assay illustrated a significant decrease in colony formation abilities of both B-CPAP and TPC-1 cells. (**C**) The effects of GSG2 knockdown on migration potential were assessed through Transwell assay. (**D**) The changes in B-CPAP and TPC-1 cell apoptosis following GSG2 depletion were analyzed through flow cytometry analysis. (**E**, **F**) Xenograft tumor models were established via subcutaneously injecting shCtrl/shGSG2-transfected TPC-1 cells, and tumor volume (**E**) and weight (**F**) were measured. Tumor volume=π/6×L×W×W. (**G**) Tumors were collected and photographed. (**H**) Immunohistochemical staining of Ki67 in mice tissues from the shCtrl and shGSG2 groups. Results were presented as mean ± SD. ** *P* < 0.01, ***P < 0.001.

The functional consequences of GSG2 knockdown were then explored. Knockdown of GSG2 led to a significant reduction in cell proliferation of B-CPAP and TPC-1, especially the downregulation of GSG2 caused by shGSG2-1 (*P* < 0.001, Figure 2A). Consistent results were observed in both B-CPAP and TPC-1 cells, where GSG2 depletion resulted in decreased colony formation (*P* < 0.001, Figure 2B). To investigate the effects on cell migration, Transwell assay was performed, revealing that GSG2-deficient cells exhibited reduced migration potential (*P* < 0.01 for B-CPAP cells and *P* < 0.001 for TPC-1 cells, Figure 2C). Furthermore, flow cytometry analysis indicated an acceleration of cell apoptosis upon knocking down GSG2 (*P* < 0.001, Figure 2D). These findings collectively demonstrated the specific and inhibitory effects of shGSG2-1 and shGSG2-2 on GSG2 expression, elucidating their role in suppressing thyroid cancer cell proliferation, colony formation, migration, and promoting apoptosis. On the other hand, to substantiate the observed *in vitro* inhibition of tumor cell growth, we conducted subcutaneous inoculation of TPC-1 cells with shCtrl and shGSG2-1 into immune-deficient mice and monitored tumor development. Consistent with the *in vitro* results, primary tumors originating from GSG2-deficient cells grew more slowly compared to those derived from shCtrl-transfected cells, as evidenced by reductions in tumor volume, weight, and size (Figure 2E–2G). In the shGSG2 group of mice tissues, although the Ki67 staining appeared weak, it is crucial to note that positive staining remained discernible, albeit with reduced intensity (Figure 2H). These collective findings provided compelling evidence that GSG2 played a role in enhancing thyroid cancer development both *in vitro* and *in vivo*.

### The exploration of downstream mechanism

**Figure 3:**
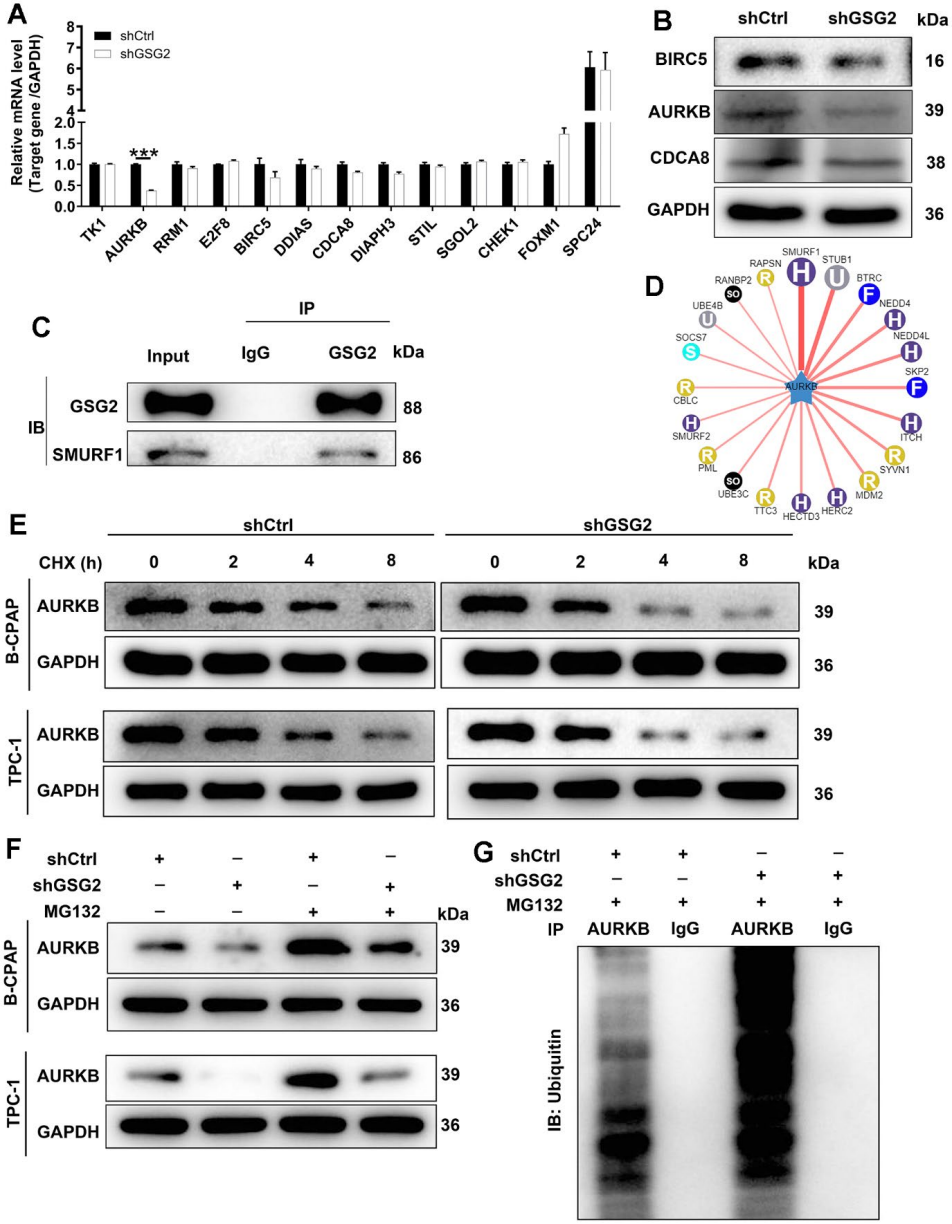
**The exploration of downstream mechanism of GSG2-regulating thyroid cancer.** (**A**) The mRNA levels of top 13 co-expressed genes of GSG2 were detected through qRT-PCR experiment. (**B**) The protein levels of BIRC5, AURKB and CDCA8 were analyzed in response to GSG2 downregulation by western blot. (**C**) Co-IP assay demonstrated an endogenous interaction between GSG2 and SMURF1. (**D**) The E3 ubiquitin ligases of AURKB. (**E**) The half-life of AURKB protein in GSG2-depleted B-CPAP and TPC-1 cells was assessed at 2 h, 4 h and 8h following 0.2 mg/mL CHX treatment. (**F**) After MG-132 addition, AURKB protein levels in GSG2-depleted B-CPAP and TPC-1 cells were measured. (**G**) The ubiquitination level of AURKB was detected in GSG2-depleted B-CPAP cells after immunoprecipitation using AURKB or IgG antibodies.

In our investigation into the mechanism by which GSG2 influenced thyroid cancer development, we initially identified co-expressed genes of GSG2 through the coexpedia website (https://www.coexpedia.org/search.php). Subsequently, out of the 110 identified genes, we focused on the top 13 with the highest scores for qRT-PCR and western blot analysis. AURKB emerged as a key candidate due to its markedly decreased mRNA and protein expression upon GSG2 depletion (Figure 3A, 3B). The levels of AURKB were also analyzed in normal cells (Nthy-ori 3-1) and thyroid cancer cell lines (CAL-62, B-CPAP, and TPC-1). The results revealed a significant increase in AURKB at the protein and mRNA levels in thyroid cancer cell lines compared to Nthy-ori 3-1 cells (Supplementary Figure 1C, 1D). Seeking to unravel the molecular interactions, we discovered that SMURF1 was an interacting protein of GSG2, forming an endogenous interaction with GSG2 (Figure 3C). Intriguingly, through the UbiBrowser website (http://ubibrowser.ncpsb.org.cn/ubibrowser/strict/networkview/networkview/name/Q96GD4/jobId/ubibrowse-I2021-07-13-25276-1626144004), we identified SMURF1 as one of the E3 ubiquitin ligases of AURKB (Figure 3D). Subsequent experiments involved assessing the half-life of AURKB protein in GSG2-deficient B-CPAP and TPC-1 cells after the addition of CHX, a protein synthesis inhibitor. The results indicated a shortened half-life of AURKB protein, suggesting accelerated AURKB degradation (Figure 3E). However, treatment with MG-132, a proteasome inhibitor, partially reversed the impacts of depleting GSG2 on the degradation of AURKB protein (Figure 3F), implying that GSG2 may regulate AURKB through the ubiquitin-proteasome system (UPS). Considering the well-established role of ubiquitination in proteasome-mediated degradation [17, 18], we further evaluated the ubiquitination level of AURKB. As depicted in Figure 3G, GSG2 knockdown significantly increased the ubiquitination level of AURKB, providing additional insights into the regulatory mechanisms of GSG2-mediated thyroid cancer.

### GSG2/AURKB participates in the development of thyroid cancer

**Figure 4:**
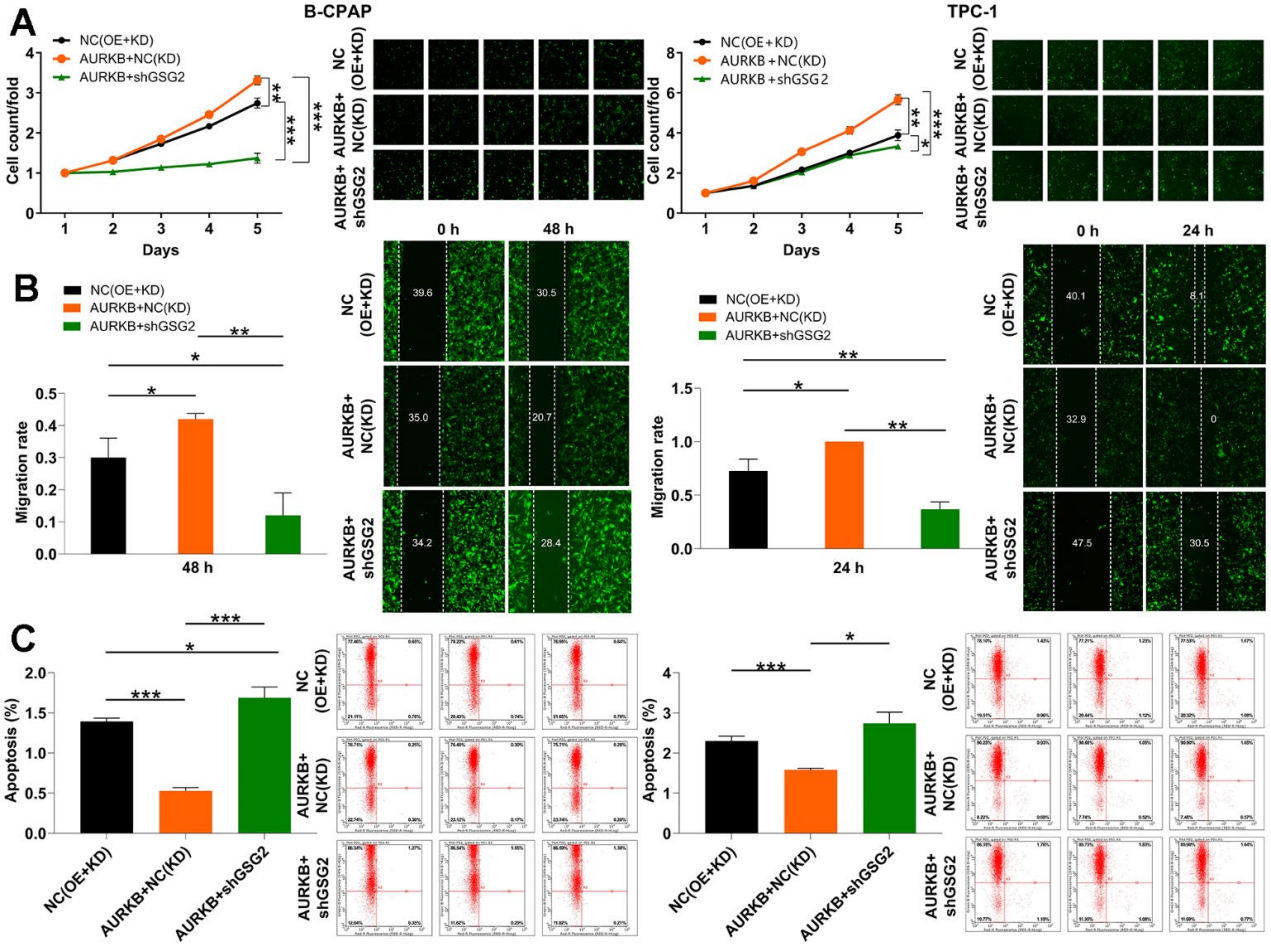
**GSG2/AURKB participates in the development of thyroid cancer.** (**A**–**C**) The alterations in cell proliferation (**A**), migration (**B**) and apoptosis (**C**) were evaluated in B-CPAP and TPC-1 cells with indicated lentiviruses through Celigo cell counting assay, wound-healing assay and flow cytometry analysis. Results were presented as mean ± SD. * *P* < 0.05, ** *P* < 0.01, ***P < 0.001; NC(OE+KD): Control, AURKB+NC(KD): AURKB overexpression, AURKB+shGSG2: AURKB overexpression+ GSG2 downregulation.

In our pursuit to comprehend the impact of GSG2 on thyroid cancer cells and ascertain whether these changes were orchestrated by AURKB, we conducted experiments. To this end, we generated lentiviral vectors for the sole overexpression of AURKB and for the simultaneous overexpression of AURKB along with the silencing of GSG2. These vectors were then used to transfect B-CPAP and TPC-1 cells. The Celigo cell counting assay data revealed that cells abundant in AURKB exhibited a heightened potential for proliferation. Furthermore, the overexpression of AURKB successfully restored the impaired proliferative activity resulting from GSG2 silencing (Figure 4A). This phenomenon was consistently observed in cell migration assays as well (Figure 4B).

Turning our attention to apoptosis, the increased AURKB led to a trend of diminished apoptotic capacity. Notably, this elevation in AURKB expression simultaneously counteracted the effects of GSG2 knockdown on cell apoptosis (Figures 4C). Overall, our findings supported the notion that the interplay between GSG2 and AURKB actively contributes to the development of thyroid cancer.

### GSG2 regulates thyroid cancer through AKT pathway

**Figure 5:**
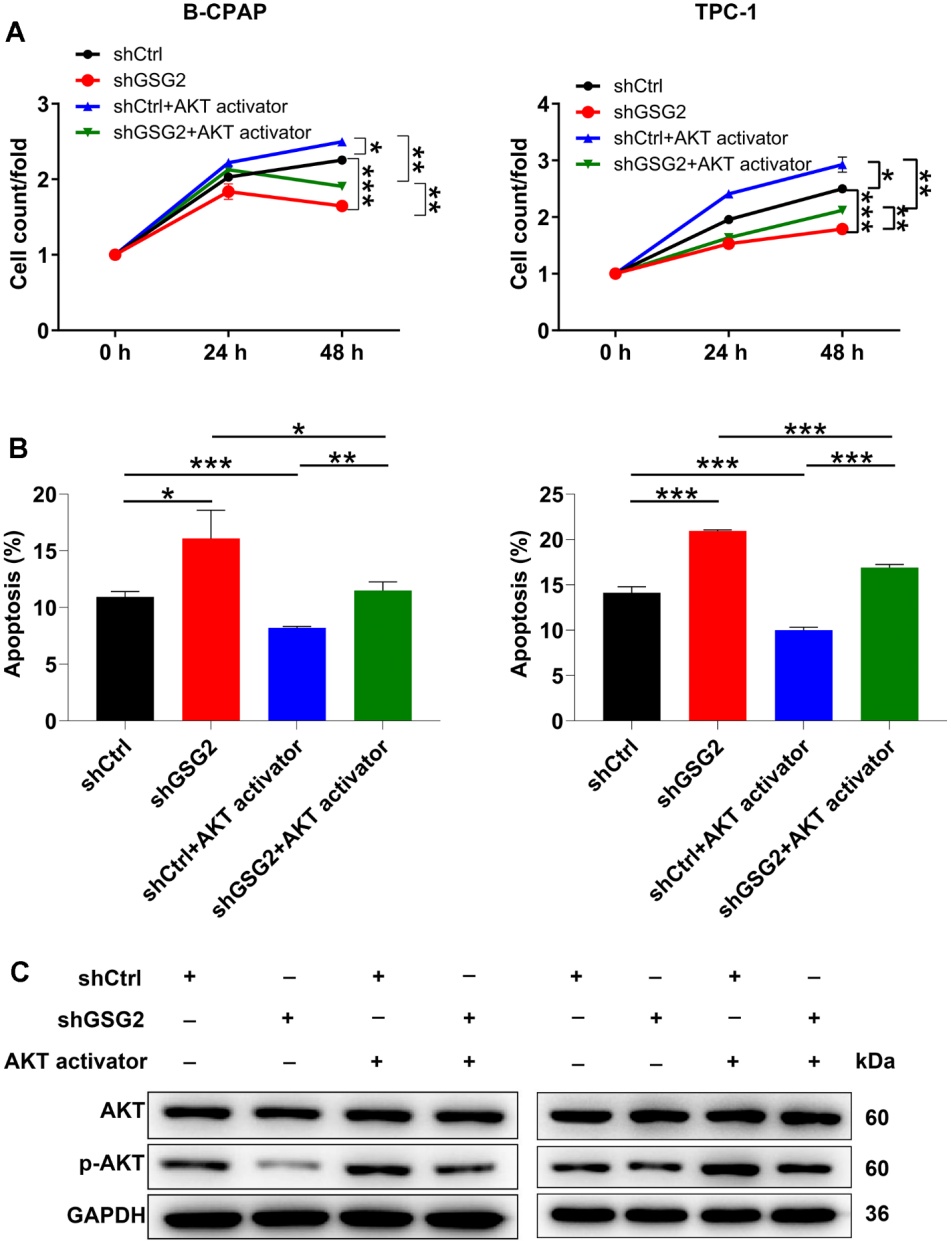
**The exploration of downstream pathway of GSG2-regulating thyroid cancer.** (**A**, **B**) After treating GSG2-depleted B-CPAP and TPC-1 cells with AKT activator, cell proliferation (**A**) and apoptosis (**B**) were assessed via CCK8 assay and flow cytometry experiments. (**C**) Total protein and phosphorylation levels of AKT were determined in GSG2-depleted B-CPAP and TPC-1 cells following AKT activator addition. Results were presented as mean ± SD. * *P* < 0.05, ** *P* < 0.01, ***P < 0.001.

In addition to exploring downstream genes, we conducted a preliminary analysis to predict the downstream pathways influenced by GSG2. The AKT pathway, known for its crucial role in normal cellular processes, has also been widely implicated in the progression of thyroid cancer [19, 20]. Notably, a previous study has unveiled the connection between GSG2 and the AKT pathway [21]. Building upon this, we hypothesized that the AKT pathway might play a role in GSG2-mediated thyroid cancer. To investigate this hypothesis, we treated GSG2-deficient B-CPAP and TPC-1 cells with an AKT activator. Remarkably, the addition of the AKT activator reversed the effects of GSG2 silencing on cell behaviors, as evidenced by enhanced cell vitality and decreased apoptosis levels (Figure 5A, 5B). Moreover, at the molecular level, GSG2 downregulation did not alter the total protein level of AKT, but it led to a decrease in its phosphorylation level (p-AKT), a change that was effectively counteracted after AKT activator treatment (Figure 5C). In summary, our findings suggested that GSG2 might regulate thyroid cancer through AKT pathway.

## DISCUSSION

In our current investigation, we observed a higher expression of GSG2 in thyroid cancer tissues compared to adjacent normal tissues. Moreover, patients with elevated GSG2 levels exhibited a more advanced pathological stage and shorter progression-free survival (PFS). Notably, the study by Koch et al. highlights the crucial role of GSG2 in overcoming resistance to EGFR inhibition by Gefitinib in A431 cells, underscoring its potential significance in combination therapies targeting this interaction [22]. Additionally, Chen et al.’s research establishes GSG2 (Haspin) as a synthetic lethal target in breast cancer. Their findings demonstrate that depleting or inhibiting GSG2 significantly enhances the therapeutic effects of MLN8237, offering a promising strategy to overcome drug resistance [23]. This cumulative evidence strongly supported our assertion that GSG2 may play a pivotal role in mediating drug resistance, urging further exploration and discussion in the context of thyroid cancer. Furthermore, our experimental data revealed that knocking down GSG2 in thyroid cancer cells led to the suppression of both *in vitro* development and *in vivo* outgrowth of thyroid cancer. These observations collectively suggested a crucial role of GSG2 overexpression in the carcinogenic processes of thyroid cancer. This reinforced the need for a comprehensive understanding of the implications of GSG2 in thyroid cancer progression and opens avenues for potential therapeutic interventions targeting GSG2-mediated drug resistance.

On the other hand, we unraveled the molecular mechanism behind GSG2 regulating thyroid cancer, identifying AURKB as a downstream gene of GSG2. Our findings indicated that GSG2 knockdown counteracted SMURF1-mediated ubiquitination of AURKB, thereby stabilizing AURKB expression at the protein level. AURKB, a protein belonging to the mitotic protein kinase family and encoded by the AURKB gene on chromosome 17, plays a pivotal regulatory role in mitosis and serves as the enzymatic core of the chromosome passenger complex [24]. Accumulated evidence demonstrates that AURKB is overexpressed in various tumors, contributing to the development and progression of malignant tumors. For instance, Nie et al. revealed that AURKB promotes gastric tumorigenesis by upregulating CCND1 expression [25]. Additionally, Bertran et al.’s study provided evidence linking AURKB elevation to non-small cell lung cancer resistance to EGFR therapy [26]. Pan et al. demonstrated that AURKB serves as a biomarker controlling cancer stem cell characteristics in bladder cancer [27]. Furthermore, AURKB was found to be expressed at higher levels in clear cell renal cell carcinoma (ccRCC) tissues, suggesting its potential as a promising biomarker in ccRCC [28]. Consistent with these findings, our study highlighted AURKB as a cancer-promoting factor in thyroid cancer, where elevated AURKB enhanced cell growth and migration capabilities. Importantly, overexpressing AURKB could rescue impaired cell functions resulting from GSG2 silencing. Given its overexpression in various tumors, AURKB has emerged as an attractive drug target [29]. Several small molecule inhibitors, such as Hesperadin, Barasertib, and SP-96, have been designed to specifically inhibit AURKB function in various tumors [30–32]. The effectiveness of these molecular inhibitors in thyroid cancer warrants further investigation in future studies.

Another noteworthy finding was that GSG2 appeared to induce thyroid development by activating the AKT signaling pathway. AKT, also known as PKB, functions as a serine/threonine protein kinase in the PI3K-AKT signaling pathway. Upon activation by PI3K, AKT exhibited the ability to suppress apoptosis and promote metastasis and invasion in malignancies [33]. The AKT pathway is recognized as one of the most crucial signaling pathways involved in normal cellular processes, and its abnormal activation is known to regulate autophagy [34], epithelial-mesenchymal transition (EMT) [35], and apoptosis [36]. Numerous published documents have highlighted the significance of the AKT signaling pathway as a key player in the progression of various malignant tumors, including thyroid cancer. For example, LINC00893 was reported to arrest thyroid cancer cell growth and migration via the AKT pathway [19]. Furthermore, Kang et al. suggested that PINX1 induces the malignant development of thyroid cancer by activating the AKT pathway [37]. Additionally, the mechanism of some targeted therapies for thyroid cancer involves the inhibition of the AKT pathway [38]. Our study demonstrated that the expression of p-AKT was downregulated by the knockdown of GSG2, and this effect was partially reversed after treatment with an AKT activator. Based on these findings, we hypothesized that GSG2 depletion might inhibit the malignant progression of thyroid cancer through the regulation of the AKT signaling pathway.

In conclusion, GSG2 played a crucial role in thyroid carcinogenesis. The observed overexpression of GSG2 in thyroid carcinoma suggested its potential as a target for the treatment of thyroid cancer. However, there are some limitations to this study. Our investigation primarily focused on elucidating the protein-protein interactions integral to the GSG2-AURKB axis. Nevertheless, we acknowledge the significance of addressing the mRNA regulatory aspects of GSG2. Moving forward, we are committed to a thorough exploration of GSG2’s multifaceted regulatory mechanisms in the future. This commitment will undoubtedly contribute to the refinement and extension of our research in subsequent studies, providing a more comprehensive understanding of GSG2’s role in thyroid cancer and potentially uncovering additional avenues for treatment.

## MATERIALS AND METHODS

### Bioinformatics analysis

We investigated the differential expression of GSG2 in thyroid cancer using the GSE29265 database, encompassing samples from both thyroid cancer and normal tissues. The data was obtained from the Gene Expression Omnibus (GEO) database (https://www.ncbi.nlm.nih.gov/geo/download/?acc=GSE29265&amp;format=file). To evaluate the prognostic significance of GSG2, we conducted an analysis on thyroid cancer samples sourced from the TCGA database, accessed through cBioPortal (https://www.cbioportal.org/). Rigorous data curation and organization were applied to the clinical information, and RNA sequencing data underwent transformation using the log2(x+1) method, where ‘x’ represents the RSEM value. Employing the median expression level of GSG2 in thyroid cancer samples as a threshold, we categorized the samples into high and low expression groups. Subsequently, a log-rank test was performed to examine the disparity in patients’ survival between these groups.

### Tissues samples and cell lines

In this study, a thyroid cancer tissue microarray (Cat. #TH801a), obtained from Xi'an Alenabio Co., Ltd. (Xi'an, China) and comprising specimens from 40 thyroid cancer patients, was utilized.

Normal thyroid cells (Nthy-ori 3-1) and three human thyroid cancer cell lines CAL-62, B-CPAP, and TPC-1 were procured from the American Type Culture Collection (ATCC) (https://www.atcc.org/). The normal thyroid cells (Nthy-ori 3-1), B-CPAP, and TPC-1 were cultured in 1640 medium supplemented with 10% FBS. CAL-62 cells were grown in DMEM supplemented with 10% FBS. All cells were maintained in a 37° C incubator with 5% CO_2_.

### Immunohistochemistry (IHC) staining

The paraffin-embedded sections underwent 1×EDTA repair (Beyotime Biotechnology Co., Ltd, Shanghai, China) and were subsequently treated with 3% H_2_O_2_ for 5 min for blocking. Following this, the tissues were incubated with primary antibodies overnight at 4° C, followed by the application of secondary antibodies as described earlier. The immunoreactivity was then assessed by introducing 3,3′-diaminobenzidine substrate (DAB, Sigma-Aldrich) and hematoxylin (Baso Diagnostics Inc., Zhuhai, China). The slides were sealed using neutral resin (China National Pharmaceutical Group Co., Ltd, Beijing, China). Finally, images were captured under an optical microscope and independently analyzed by three pathologists in a randomized manner. Positive cell scores were categorized as follows: 1 (1%-24%), 2 (25%-49%), 3 (50%-74%), and 4 (75%-100%). Staining intensity received scores of 0 (Signalless color), 1 (light yellow), 2 (brown yellow), and 3 (dark brown). The IHC results were determined by multiplying the positive cell score by the staining color intensity score, with a higher score indicating elevated antibody expression. The scoring system was defined as follows: 0 (negative), 1-4 (positive), 5-8 (positive++), 9-12 (positive+++). The criteria for high and moderate expression were established based on the median of IHC scores derived from all tissue samples. Samples with scores surpassing the median were categorized as high expression, while those with scores below the median were classified as low expression. Details of the antibodies used can be found in Supplementary Table 1.

### Small interfering RNA, overexpression plasmid and plasmid infection

Using GSG2 as a template, RNA interference target sequences were designed for the construction of short hairpin RNA expressing GSG2 (shGSG2) lentiviral vectors. The specific RNAi sequences were as follows: RNAi 10055 (CCACAGGACAATGCTGAACTT) and RNAi 00177 (AGGGATTGACTTAGAGCAAAT). As a negative control, a scramble sequence (TTCTCCGAACGTGTCACGT) was employed. The AURKB overexpression plasmid was meticulously constructed by BIOSCIRES Co., Ltd. (Shanghai, China). In the process of plasmid transfection, 2×10^5^ B-CPAP and TPC-1 cells were subjected to lentiviral particle transfection, utilizing a concentration of 1×10^8^ TU/mL, under ENI.S+Polybrene conditions.

### RNA extraction, cDNA synthesis and quantitative real-time PCR (qRT-PCR)

Total RNA was extracted using TRIzol reagent (Sigma-Aldrich, St. Louis, MO, USA). Subsequently, cDNA was synthesized through reverse transcription, employing 2.0 μg of RNA, following the manufacturer’s instructions provided in the Promega M-MLV Kit (Promega, Heidelberg, Germany). qRT-PCR was conducted on the Biosystems 7500 Sequence Detection system using the SYBR Green Master Mix Kit (Vazyme, Nanjing, Jiangsu, China). GAPDH served as an internal normalization control. The relative expression of mRNA was assessed utilizing the 2^-∆∆Ct^ method. The primer sequences (5′-3′) were detailed in Supplementary Table 2.

### Western blot assay and co-immunoprecipitation (Co-IP)

Total protein was extracted using the BCA Protein Assay Kit (HyClone-Pierce, Cat. No. 23225). Following protein extraction, PVDF membranes were blocked for 1 h using a TBST solution containing 5% skim milk. The proteins were then separated by 10% SDS-PAGE and transferred to PVDF membranes for immunostaining. Then, the membranes underwent incubation with primary and secondary antibodies at room temperature for 2 h. The color rendering process was performed using the immobilon Western Chemiluminescent HRP Substrate kit (Millipore, Cat. No. RPN2232).

For the Co-IP assay, proteins from B-CPAP cells were immunoprecipitated using GSG2 and IgG antibodies. The immunoprecipitated proteins were then subjected to immunoblotting with antibodies specific to GSG2 and SMURF1. Details of all antibodies used could be found in Supplementary Table 1.

### Analyses of protein degradation

B-CPAP and TPC-1 cells with depleted GSG2 were treated with 0.2 mg/mL cycloheximide (CHX) and harvested at 2 h, 4 h, and 8 h. The cell lysates obtained were then subjected to immunoblotting with an AURKB antibody to assess protein levels. For the analysis of AURKB ubiquitination levels, cells were co-transfected with AURKB and ubiquitin. After 24 h of infection, the proteasome inhibitor MG132 (10 μM) was introduced. Subsequently, cell lysates were immunoprecipitated with AURKB or IgG antibody overnight at 4° C. Protein A/G Plus–agarose was added for 4 h at 4° C, followed by immunoblotting using an ubiquitin antibody. The specific antibodies used in these experiments were detailed in Supplementary Table 1.

### Cell proliferation detection

In this study, the cell proliferation ability was assessed through CCK-8 detection and Celigo cell counting assay. For the CCK-8 detection, B-CPAP and TPC-1 cells, post lentiviral transfection and/or treated with a 20 μM AKT activator, were subjected to the same procedure as described above. The next day, CCK-8 reagent was added for 4 h, and the OD value was detected using a microplate reader at 450 nm.

In the Celigo cell counting assay, B-CPAP and TPC-1 cells were treated as mentioned earlier. Cell images were captured using the Celigo image cytometer (Nexcelom Bioscience, Lawrence, MA, USA), and a continuous 5-day cell proliferation curve was generated.

### Colony forming assay

B-CPAP and TPC-1 cells, transfected with the specified lentivirus, were seeded in a 6-well plate at a density of 500 cells per well and cultured for 8 days. Following the culture period, the colonies were fixed with 1 mL of 4% paraformaldehyde and stained using 500 μL of Giemsa (Dingguo, Shanghai, China). Visible clones were documented using a fluorescence microscope (Olympus, Tokyo, Japan).

### Cell migration detection

Cell migration changes were assessed through both the Wound Healing assay and the Transwell assay. The specific procedure for the former is detailed as follows: B-CPAP and TPC-1 cells, transfected with the specified lentivirus, were cultured in a 96-well plate at a density of 5×10^4^ cells per well. After 24 h, a low-concentration serum medium was introduced, and scratches were created using a scratch tester. Subsequently, the cells were incubated in a 5% CO_2_ environment at 37° C. At an appropriate time, the plate was scanned, and the migration area was analyzed using Cellomics (Thermo, USA).

The latter assay was performed as outlined below: B-CPAP and TPC-1 cells, also transfected with the indicated lentivirus at a density of 5×10^4^, were seeded on a fibronectin-coated polycarbonate membrane inserted into a Transwell apparatus (Costar, MA). In the lower chamber, 500 μL of medium with 10% FBS served as a chemoattractant. Finally, the cells were stained with a 1% crystal violet solution for 1 min, and the number of cells was counted under a microscope in three random fields.

### Fluorescence activated cells sorting (FACS)

For the analysis of changes in cell apoptosis, both double staining and single staining methods were employed. In the double staining approach, lentivirus-transfected cells were cultured in a 6-well plate with 2 mL per well. Subsequently, the cells were washed with D-Hanks (4° C, pH=7.2~7.4) and stained in the dark by adding 5 μL Annexin V-APC and 5 μL propidium iodide (PI). The level of cell apoptosis was assessed using the FACSCalibur flow cytometer (BD Biosciences, San Jose, CA, USA).

As for the single staining method, B-CPAP and TPC-1 cells with the specified lentivirus were cultured in a 6-well plate with a volume of 2 mL per well. Following this, the cells were washed with D-Hanks (4° C, pH=7.2~7.4) and stained in the dark by adding 10 μL Annexin V-APC (eBioscience, San Diego, CA, USA). The FACSCalibur (BD Biosciences, San Jose, CA, USA) was utilized to evaluate the level of cell apoptosis.

### The construction of nude mouse tumor formation model

The *in vivo* experiments were ethically approved by both the Ethics Review Committee of Guangzhou Medical University and the Ethics Review Committee of Guangdong Provincial People’s Hospital. Female BALB/c nude mice, aged 4 weeks, were provided by Jiangsu Jicui Yaokang Biotechnology Co., Ltd. The mice were housed under specific conditions: 5 mice per cage, temperature maintained at 22-25° C, humidity at 50-60%, and a 12-h light/dark cycle. Adequate water and food supplies were provided to ensure free access for the mice. Xenograft models were established by subcutaneously injecting GSG2-depleted TPC-1 cells (1 × 10^7^ cells/each) into the right axilla of nude mice (5 mice/group). Tumor dimensions, including length (L) and width (W), were measured, and tumor volume was calculated using the formula: tumor volume = π/6 × L × W × W. This assessment was carried out over a period of 25 days. After the experimental period, the mice were euthanized, and the tumors were excised and weighed.

### Statistical analysis

The data obtained from a minimum of three repeated experiments were subjected to analysis using GraphPad Prism 6 (San Diego, CA, USA) and SPSS 19.0 software. The results were presented in the form of mean ± standard deviation (SD). Statistical significance was assessed through Student’s t-test for comparisons involving two groups and one-way ANOVA for multiple group comparisons. To explore the relationship between GSG2 expression and pathological characteristics of thyroid patients, Spearman correlation analysis and Mann-Whitney U analysis were employed. The threshold for statistical significance was set at a *P*-value less than 0.05.

### Availability of data and materials

The data generated in this study are available within the article and its Supplementary Data File 1.

## Supplementary Material

Supplementary Figure 1

Supplementary Tables

Supplementary Data File 1
