# Supplementary material for: GSG2 promotes thyroid cancer via stabilizing AURKB and activating AKT pathway
Source: Aging (Albany NY). 2024 Mar 4;16(6):5091–107. doi: 10.18632/aging.205605 (PMC11006493; doi:10.18632/aging.205605)
Supplement: Supplementary Figure 1 [file aging-16-205605-s001.pdf]

SUPPLEMENTARY FIGURE

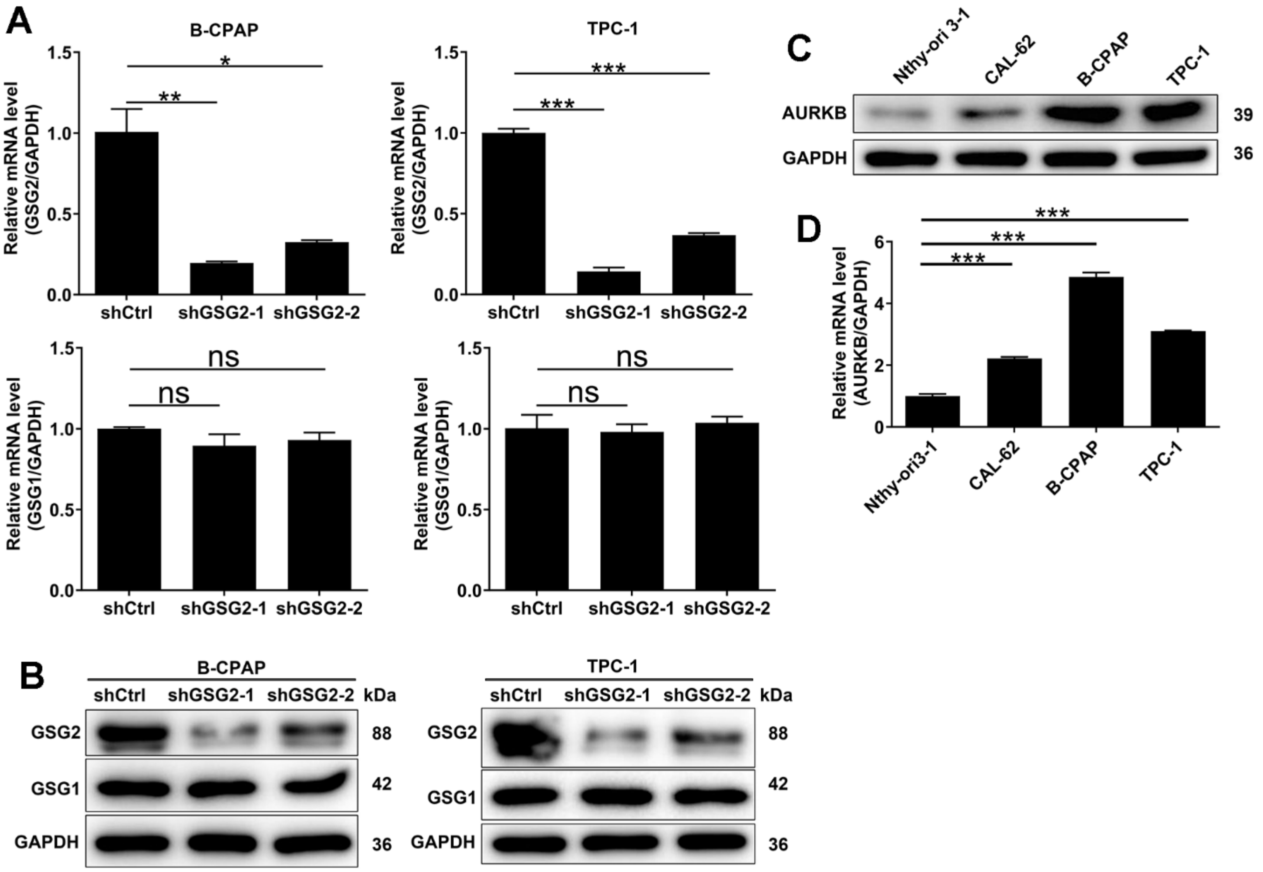

Supplementary Figure 1. (**A**, **B**) GSG1 and GSG2 levels were evaluated in B-CPAP and TPC-1 cells transfected with shGSG2-1 and shGSG2-2, as assessed by qRT-PCR (**A**) and western blot analyses (**B**). (**C**, **D**) The protein (**C**) and mRNA (**D**) levels of AURKB in thyroid cancer cell lines (CAL-62, B-CPAP, and TPC-1) and normal cells (Nthy-ori 3-1) were analyzed through western blot and qRT-PCR. Results were presented as mean ± SD. * *P* < 0.05, ** *P* < 0.01, ****P* < 0.001.
