# Supplementary material for: GSG2 promotes thyroid cancer via stabilizing AURKB and activating AKT pathway
Source: Aging (Albany NY). 2024 Mar 4;16(6):5091–107. doi: 10.18632/aging.205605 (PMC11006493; doi:10.18632/aging.205605)
Supplement: Supplementary Tables [file aging-16-205605-s002.pdf]

SUPPLEMENTARY TABLES

Supplementary Table 1. Antibodies used in Western blot and IHC analysis.

Primary antibodies	Dilution in WB	Source species	Company	Catalog no.
GSG2	1:1000	Rabbit	Abcam	ab21686
BIRC5	1:2000	Rabbit	Proteintech	10508-1-AP
AURKB	1:500	Mouse	Santa Cruz	sc-393357
CDCA8	1:1000	Rabbit	Abcam	ab74473
SMURF1	1:500	Rabbit	Proteintech	55175-1-AP
AKT	1:3000	Rabbit	CST	4691S
p-AKT	1:2000	Mouse	Proteintech	66444-1-Ig
Ubiquitin	1:1000	Mouse	Santa Cruz	sc-47721
GAPDH	1:3000	Rabbit	Bioworld	AP0063
**Secondary antibody**	**Dilution**		**Company**	**Catalog no.**
Goat Anti-Rabbit	1:3000		Beyotime	A0208
Goat Anti- Mouse	1:3000		Beyotime	A0216
**Primary antibodies**	**Dilution in IHC**	**Source species**	**Company**	**Catalog no.**
GSG2	1:200	Rabbit	Bioss	BS-15413R
Ki67	1:100	Rabbit	Abcam	Ab16667
**Secondary antibody**	**Dilution**		**Company**	**Catalog no.**
Goat Anti-Rabbit	1:400		Abcam	A6721

Supplementary Table 2. Primers used in qRT-PCR.

Gene	Forward primer sequence (5′-3′)	Reverse primer sequence (5′-3′)
GSG2	GGAAGGGGTGTTTGGCGAAGT	TGAGGAGCAAGGGAGGGTAAG
TK1	CAAGTATGCCAAAGACACTCGCTAC	AACTGCCCCTCGTCGATGCC
AURKB	CATGGAGGAGTTGGCAGATG	CCTTGAGCCCTAAGAGCAGAT
RRM1	GAAAGAGCAACCAGCAGAACC	GGGAAGCCAAATTACAAACAGC
E2F8	CCTGAGATCCGCAACAGAGAT	AGATGTCATTATTCACAGCAGGG
BIRC5	TCTCAAGGACCACCGCATCT	TTTGCATGGGGTCGTCATCT
DDIAS	GAAGGGGACATTATCTGG	TTCAGCAAACACCAACAT
CDCA8	GCCAAGATGAGACGGGATGAC	TTCCTCCAAGGGCGAAGTAGTC
DIAPH3	TTTACCGACCACCAAGAA	GGCAAGGCACCAAACTGA
STIL	CGGTTCCTCACAGAACAAGACA	TAGGGGAACAGGGCATCAGA
SGOL2	TGAGATGAGAAACGCCCAGTC	TTCCCAAGATGACCCACGCT
CHEK1	TTGGCTTGGCAACAGTATTTCG	CCAGCGAGCATTGCAGTAAGT
FOXM1	AGTTCCCGGTGAACCAGTCA	ACACCACCTGTTCCCCAAA
SPC24	TGGTCAAAGGCATCCATCA	GACTCCAGAGGTAGTCGCTGAT
GAPDH	TGACTTCAACAGCGACACCCA	CACCCTGTTGCTGTAGCCAAA
